# Effect of Er,Cr (YSGG Laser Root Conditioning on the Success of Root Coverage with Subepithelial Connective Tissue Graft): A Randomized Clinical Trial with a 6-Month Follow-Up

**Published:** 2018-07

**Authors:** Banafsheh Poormoradi, Parviz Torkzaban, Leila Gholami, Amirarsalan Hooshyarfard, Maryam Farhadian

**Affiliations:** 1 Assistant Professor, Department of Periodontics, School of Dentistry, Hamadan University of Medical Sciences, Hamadan, Iran; 2 Associate Professor, Department of Periodontics, School of Dentistry, Hamadan University of Medical Sciences, Hamadan, Iran; 3 Postgraduate Student, Department of Periodontics, School of Dentistry, Hamadan University of Medical Sciences, Hamadan, Iran; 4 Assistant Professor, Department of Biostatistics, School of Public Health and Research Center for Health Sciences, Hamadan University of Medical Sciences, Hamadan, Iran

**Keywords:** Gingival Recession, Tooth Root, Tissue Transplantation, Lasers

## Abstract

**Objectives:**

Finding predictable approaches for root surface biomodification is an important challenge in the treatment of gingival recession. This study sought to assess the root coverage percentage by subepithelial connective tissue graft (SCTG) following root surface conditioning with erbium, chromium: yttrium scandium gallium garnet (Er,Cr:YSGG) laser.

**Materials and Methods:**

In this split-mouth, randomized clinical trial, 30 teeth with Miller’s Class I and II gingival recession were treated with SCTG (the Langer and Langer technique) with (case group) or without (control group) root surface conditioning with Er,Cr:YSGG laser (wavelength=2780 nm, power=0.75 W, H mode, repetition rate=20 Hz). Recession depth (RD), recession width (RW), clinical attachment level (CAL), and probing depth (PD) were assessed at the baseline (one week before surgery) and at 2 and 6 months postoperatively. The amount of root coverage was quantified in the two groups. Data were analyzed using Friedman test and Wilcoxon signed-rank test.

**Results:**

No significant difference was noted between the case and control groups in any parameter (P>0.05). Significant improvement occurred in all the measured parameters in the two groups after surgery (P<0.05). The mean root coverage at the end of the study period was 87% and 80% in the case and control groups, respectively (P=0.244), and complete root coverage was achieved in 66% and 60% of the samples in the case and control groups, respectively.

**Conclusions:**

Root surface conditioning by Er,Cr:YSGG laser improved the mean root coverage and the percentage of complete root coverage. However, these changes were not statistically significant.

## INTRODUCTION

Gingival recession is an unfavorable clinical condition characterized by the migration of the gingival margin from the cementoenamel junction (CEJ) towards the apex [1]. It results in denuding of the root surface and subsequent tooth hypersensitivity, root caries, and esthetic problems [2–4].

A high percentage of adults suffer from gingival recession [5]. Inflammatory periodontal disease, shortage of keratinized tissue, mechanical trauma, orthodontic movement, buccal positioning of the root, bone dehiscence, and abnormal frenal attachment are among the causes of gingival recession [5].

The main goal of treatment is to cover the denuded root surface to decrease tooth hypersensitivity and improve esthetics. Different therapeutic approaches suggested for this condition include surgical and non-surgical treatment modalities. Surgical approaches include free gingival graft [6], subepithelial connective tissue graft (SCTG) [7], coronally advanced flap [8], laterally sliding flap [9], double papillae flap [10], guided tissue regeneration [11], and acellular dermal matrix allograft [12]. The success rate of these procedures depends on several factors such as the position of the tooth, the class of recession, surgeon’s experience and expertise, the surgical technique, and postoperative care [6–14]. Of the aforementioned procedures, SCTG has shown a high success rate and optimal predictability [13–15]. Thus, SCTG is considered the gold standard for the assessment of novel approaches [16].

Several studies have evaluated the efficacy of root surface conditioning aiming to improve the treatment results. It has been reported that mechanical debridement preserves the smear layer on the root surface and thus, prevents cell reattachment to this surface, compromising the process of regeneration and repair [17]. Several strategies have been proposed to overcome this problem including the use of root surface conditioners such as citric acid [18], ethylenediaminetetraacetic acid (EDTA) [19], tetracycline hydrochloride [20], and hydrogen peroxide [21], enamel matrix proteins [22], platelet-rich plasma [23], and recombinant human growth factors [24].

A recent study showed that laser irradiation eliminates the smear layer and exerts bactericidal effects and can therefore improve the condition of root surface for connective tissue attachment [25]. In contrast to carbon dioxide (CO_2_) and neodymium-doped yttrium aluminum garnet (Nd:YAG) lasers that have limited applications only for the soft tissue, erbium-doped yttrium aluminium garnet laser (Er:YAG; 2940 nm) and erbium, chromium: yttrium scandium gallium garnet (Er,Cr:YSGG; 2780 nm) laser are suitable for use on hard tissues [26]. To date, published information about the clinical outcome of the application of Er,Cr:YSGG laser for the treatment of gingival recession is scarce. Thus, this study aimed to assess and compare the clinical results of gingival recession treatment by SCTG with/without root surface conditioning with Er,Cr:YSGG laser.

## MATERIALS AND METHODS

This split-mouth, randomized clinical trial has been approved by the ethics committee of Hamadan University of Medical Sciences (IR.UMSHA.REC.1396.115) and is registered at the Iranian Registry of Clinical Trials (IRCT201705309014N167).

### Patient selection:

The patients were selected from among those presenting to the Periodontics Department of School of Dentistry, Hamadan University of Medical Sciences with gingival recession defects. The study was thoroughly explained to the patients, and written informed consent was obtained from them.

### The inclusion criteria:

1. Good oral hygiene (plaque index <30%)
2. Miller’s Class I and II gingival recession defects [27]
3. No tooth mobility
4. Absence of trauma from occlusion

### The exclusion criteria:

1. Positive medical history contraindicating dental interventions
2. Presence of coagulation problems
3. Intake of medications interfering with periodontal health or the healing process
4. Alcohol consumption, tobacco use, or cigarette smoking
5. Disability or not showing up for follow-up sessions

Scaling, root planing, and crown polishing were performed for all patients four weeks prior to surgery. Oral hygiene instructions were also given to the patients. To ensure the absence of periapical lesions, a parallel periapical radiograph was taken from the respective teeth, and the teeth were then randomly divided into two groups of case and control.

### Assessment of clinical parameters:

The following clinical parameters were measured at the buccal surface of the teeth one week prior to surgery and at 2 and 6 months postoperatively using a periodontal probe (Williams Periodontal Probe, Hu-Friedy, Chicago, IL, USA):

- Recession depth (RD): From the CEJ to the lowest point of migration of the gingival margin.
- Recession width (RW): Distance from the mesial to the distal aspect of the gingival margin at the level of the CEJ.
- Clinical attachment level (CAL): From the CEJ to the bottom of the gingival sulcus.
- Probing depth (PD): From the gingival margin to the bottom of the gingival sulcus.

### Calibration of the examiner:

The intraclass correlation coefficient was calculated for the assessment of the reproducibility of measurements, which was found to be 0.99, and indicated an excellent intraobserver agreement.

### Treatment protocol:

For all patients, 10% povidone-iodine (Iran Najo Pharmaceutical Hygienic & Cosmetic Co., Tehran, Iran) and 0.2% chlorhexidine (Iran Najo Pharmaceutical Hygienic & Cosmetic Co., Tehran, Iran) were used for extraoral and intraoral disinfection, respectively. Lidocaine plus epinephrine (Persocaine-E^®^, Darou Pakhsh Pharmaceutical Mfg. Co., Tehran, Iran) was used for local anesthesia.

Gingival recession defects were treated according to the Langer and Langer technique [7]. A partial-thickness flap was elevated with two vertical incisions wider than the recession area by the length or half-length of a tooth mesiodistally. The coronal margin of the flap was prepared by a sulcular horizontal incision.

Interdental papilla remained untouched. The flap was extended to the mucobuccal fold without causing any perforation. Root surfaces were curetted to eliminate irregularities and dental plaque. A proper size connective tissue graft with 2 mm thickness was harvested from the palate using the trap-door technique [14]. The area was sutured with non-resorbable stitches (Braided Silk 4-0, SUPASIL, SUPA Medical Devices Co., Tehran, Iran).

Er,Cr:YSGG laser (Waterlase; Biolase. Technologies, San Clemente, CA, USA) was used for root surface conditioning in the case group. The laser optic fiber was positioned perpendicular to the surface at a distance of 1–2 mm. The laser was irradiated at 2780 nm wavelength, 20 Hz repetition rate, 0.75 W power, H mode [28] with 60% water and 40% air, using a Gold handpiece with MZ6 tip (600 μm in diameter and 6 mm in length) in spiral motion (vertical, horizontal, and oblique directions) and defocused mode. Eyeglasses with a suitable optical density were worn.

The graft was trimmed if required and was then fixed at the recipient site using resorbable stitches (Polyglycolate coated 4-0, SUPABON, SUPA Medical Devices Co., Tehran, Iran). For better blood supply, the flap covered a large part of the graft. Eugenol-free periodontal dressing (Coe-Pak, GC America, Alsip, IL, USA) was applied on the surgical site and was repeated after one week. At the end of the second week, the stitches were removed.

The root coverage treatments in each patient were performed with an interval of 6 weeks between the first and the second surgeries.

### Postoperative care:

Amoxicillin (500 mg every 8 hours for 7 days; LOGHMAN Pharmaceutical & Hygienic Co., Tehran, Iran) and ibuprofen (400 mg every 6 hours for 48 hours; ADVIFEN^®^, ZAHRAVI Pharmaceutical Co., Tehran, Iran) were prescribed postoperatively. The patients were requested to use soft food and not to brush the teeth at the surgical site for 14 days. Also, 0.2% chlorhexidine mouthwash was prescribed twice a day, each time for one minute. The sutures were then removed, chlorhexidine was prescribed for two more weeks, and tooth brushing with a soft toothbrush was recommended twice a day. Dental prophylaxis was performed two weeks after suture removal and then monthly until the end of the study period. To ensure the absence of bias, all surgical treatments were done by a single surgeon, whereas the clinical measurements were made by another examiner.

**Fig. 1:**
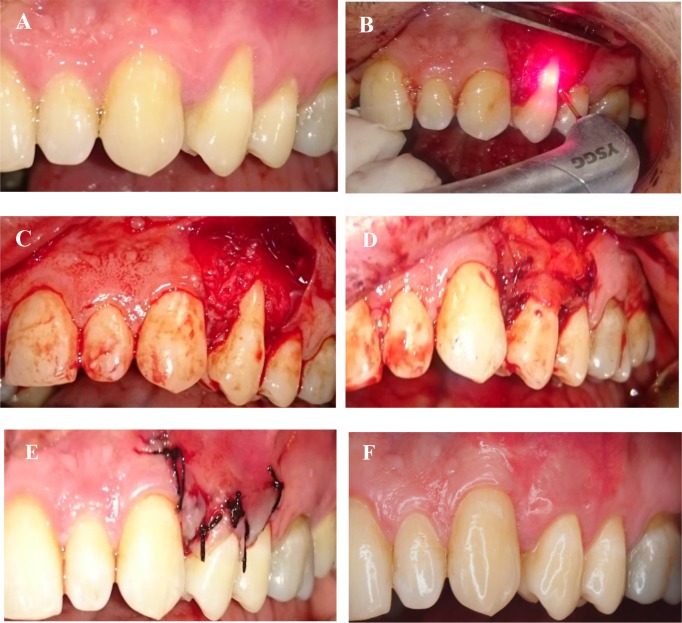
(A) Tooth #12 before treatment. (B) Elevation of a partial-thickness flap. (C) Root surface conditioning. (D) Graft placement at the recipient site. (E) Suturing the flap at the recipient site. (F) Tooth #12 after treatment

Figure 1 shows the stages of the treatment in a patient.

### Assessment of root coverage:

The percentage of root coverage was calculated using the following formula:

Root Coverage (RC) = (Preoperative RD − Postoperative RD) / Preoperative RD × 100

### Data analysis:

Data were analyzed by SPSS version 24 software program (SPSS Inc., Chicago, IL, USA). Descriptive data were reported. Since data were not normally distributed according to Kolmogorov-Smirnov test, non-parametric tests were used for data analysis. Intragroup comparisons of the variables were made using Friedman test.

In case of significant results, Wilcoxon signed-rank test with Bonferroni adjustment was applied for pairwise comparisons. Wilcoxon signed-rank test was used for intergroup comparisons. Alpha=0.05 was considered statistically significant for all comparisons, except for those requiring Bonferroni adjustment, for which, alpha=0.008 was considered significant.

## RESULTS

Five male patients with a mean age of 36.4±8.35 years were enrolled. Thirty teeth requiring treatment for gingival recession were evaluated in two groups of 15, matched in terms of the type of the tooth (anterior teeth or premolars) and the type of recession (Miller’s Class I or II). Both case and control groups included 5 anterior teeth with Miller’s Class I recession, 2 anterior teeth with Miller’s Class II recession, 3 premolars with Miller’s Class I recession, and 5 premolars with Miller’s Class II recession.

The clinical parameters were compared at the baseline (one week before surgery) and at 2 and 6 months postoperatively between the two groups (Table 1).

**Table 1:** Comparison of clinical parameters (mm) between the two groups at different time points

**Clinical parameters**	**Case group**	**Control group**	*P*-value^*^
**Recession depth (RD)**			
Baseline	3.27±0.70	3.20±0.77	0.855
2 months	0.60±0.82	0.93±1.22	0.262
6 months	0.40±0.63	0.73±0.96	0.163

**Recession width (RW)**			
Baseline	2.67±0.81	2.47±0.74	0.429
2 months	0.93±1.22	1.00±1.30	0.739
6 months	0.80±1.20	1.00±1.30	0.480

**Clinical attachment level (CAL)**			
Baseline	4.80±1.20	4.67±1.11	0.586
2 months	1.93±1.22	2.40±1.68	0.327
6 months	1.33±0.97	1.80±1.32	0.142

**Probing depth (PD)**			
Baseline	1.53±0.64	1.47±0.64	0.655
2 months	1.53±0.51	1.60±0.63	0.655
6 months	1.13±0.35	1.27±0.45	0.134

*: Wilcoxon signed-rank test

No significant difference was found between the two groups at different time points (P>0.05). In the case group, significant changes occurred in RD, RW, and CAL at different time points.

RD decreased from 3.27±0.70 mm at the baseline to 0.82±0.60 mm at 2 months and 0.63±0.40 mm at 6 months postoperatively. This reduction, according to Friedman test, was statistically significant (P<0.001). The results of Wilcoxon signed-rank test showed that the reduction in RD between 2 and 6 months was not significant (P=0.083), but the reduction in RD at 2 and 6 months compared to the baseline was statistically significant (P=0.001).

At the baseline, RW was 2.67±0.81 mm, which significantly decreased to 1.22±0.93 mm at 2 months and to 1.20±0.80 mm at 6 months postoperatively (P<0.001). The change from 2 to 6 months was not significant (P=0.317), but significant changes were noted at 2 months (P=0.001) and at 6 months (P<0.001) compared to the baseline. CAL also significantly decreased from 4.80±1.20 mm at the baseline to 1.93±1.22 mm at 2 months and 1.33±0.97 mm at 6 months postoperatively (P<0.001).

These improvements were significant at 2 and 6 months compared to the baseline (P=0.001) and also at the time interval between 2 and 6 months (P=0.007).

The same changes were recorded in the control group.

A significant reduction occurred in RD from 3.20±0.77 mm at the baseline to 1.22±0.93 mm at 2 months and 0.96±0.73 mm at 6 months postoperatively (P=0.001). This reduction during the time interval between 2 and 6 months was not significant (P=0.083), but the reduction at 2 and 6 months compared to the baseline was statistically significant (P=0.001).

RW decreased from 2.47±0.74 mm at the baseline to 1.30±1.00 mm at 2 and 6 months (P<0.001); this reduction was statistically significant compared to the baseline (P=0.001). A significant reduction occurred in CAL from 4.67±1.11 mm at the baseline to 2.40±1.68 mm at 2 months and 1.80±1.32 mm at 6 months postoperatively (P<0.001). The reduction at 2 and 6 months was significant compared to the baseline (P=0.001), but the change during the time interval between 2 and 6 months was not statistically significant (P=0.014).

PD did not experience any significant change at the mentioned time points in any group (P>0.05). No significant difference was found in root coverage between the case and control groups postoperatively (P>0.05, Table 2).

**Table 2:** Comparison of postoperative root coverage in the two groups

**Clinical parameters**	**Case group**	**Control group**	P-value^*^
**Root coverage (mm)**			
2 months	2.67±1.11	2.27±1.03	0.207
6 months	2.86±0.99	2.46±0.83	0.196

**Root coverage (%)**			
2 months	81.11±27.18	74.44±33.10	0.396
6 months	87.22±20.62	80.00±26.12	0.244

*: Wilcoxon signed-rank test

Table 3 shows the frequency of complete root coverage achieved postoperatively in the two groups.

**Table 3:** Frequency of complete root coverage achieved postoperatively in the two groups

**Complete root coverage**	**A** **N(%)**	**B** **N(%)**	**C** **N(%)**
**Case Group**			
2 Months	9 (60)	3 (20)	3 (20)
6 Months	10 (66.6)	4 (26.7)	1 (6.7)

**Control Group**			
2 Months	9 (60)	2 (13.3)	4 (26.7)
6 Months	9 (60)	5 (33.3)	1 (6.7)

Level of root coverage (A=100% B=50–99% C=0–49%)

## DISCUSSION

The current study showed that the use of Er,Cr:YSGG laser for root surface conditioning had no significant effect on the outcome of the treatment of gingival recession with SCTG. The mean CAL, RD, and RW in both groups significantly decreased during the study period. The mean root coverage in the case and control groups at 2 months postoperatively was 81% and 74%, respectively. These values were 87% and 80% at 6 months, respectively. Complete root coverage in both groups had a frequency of 60% at 2 months. This value in the case group increased to 67% at 6 months but remained unchanged in the control group. These findings are comparable to those of previous studies reporting a range of 64.7% to 97.3% for the mean root coverage and 18.1% to 96.1% for complete root coverage [16,21, 29].

The commonly used modalities for the treatment of gingival recession during the 1960s and 1970s included free gingival graft [6] and pedicle grafts [8–10]. SCTG was used for this purpose in the early 1980s [7]. Several studies have reported the high success rate and high predictability of this approach [13–15]. Thus, SCTG was used for the treatment of gingival recession in this study.

The final goal of the treatment of gingival recession is to achieve complete root coverage to improve esthetics and eliminate tooth hypersensitivity [16,21, 29]. Several conditioners have been used for this purpose, but controversy exists regarding their efficacy for the improvement of clinical parameters.

Some studies reported that conditioned root surfaces showed higher percentages of complete root coverage compared to unconditioned areas [30,31]. In contrast, the results of some other studies indicated no significant advantage for root conditioners [3,18, 20]. Our results revealed that root surface conditioning by Er,Cr:YSGG laser did not improve the clinical results of SCTG.

Published clinical data regarding the results of the application of Er,Cr:YSGG laser for the treatment of gingival recession are not available. However, the application of laser has been recommended as an adjunct for this purpose. Clinical studies have demonstrated that laser is beneficial for improving the results of regenerative treatments since it reinforces the attachment of regenerated periodontal structures [32–34]. In an in-vitro study, Fekrazad et al [28] evaluated the efficacy of Er,Cr:YSGG laser for root surface conditioning compared to EDTA and no use of conditioner. They stated that laser irradiation had a higher potential for reinforcement of the attachment of fibroblasts, while EDTA caused no significant change in the results [28].

Er:YAG laser is a promising modality for periodontal treatment [35,36]. Clinical studies have reported the optimal efficacy of erbium laser for the treatment of periodontal pockets via surgical and non-surgical techniques [37,38] and also for root conditioning [39,40].

Changes caused by the thermomechanical effects of Er:YAG laser on the root surface include a change in the microstructure as well as thermal alterations [34,41–43]. The changes occurred in surface microstructure are considered advantageous for the primary attachment of cells and tissues in the clinical setting and result in the better formation of fibrin and blood clots [41–43]. Due to high absorption in water, erbium lasers have a high power for ablation of dental hard tissues [34,35] without causing significant thermal complications such as carbonization, melting, or crack formation in the root structure, which are often seen following the use of CO_2_ and Nd:YAG lasers [34,44]. Considering the biocompatibility of the surfaces lased with Er:YAG laser, several studies have shown the better attachment and faster proliferation of fibroblasts on these surfaces compared to mechanically debrided surfaces [17,45]. The Er,Cr:YSGG laser parameters used in this study included 2780 nm wavelength, 0.75 W power, H mode, and 20 Hz repetition rate, according to the recommendations of a previous study, to preserve the biocompatibility of lased root surfaces [28].

In contrast, some authors reported that root surfaces lased with Er:YAG laser showed significant micron-scale irregularities in vitro [41,43]. Fujii et al [41] demonstrated that lased root surfaces had a specific microstructure along with denatured collagen fibers. Regarding other lasers, Trylovich et al [46] reported that the application of Nd:YAG laser changed the biocompatibility of root surfaces, making them unsuitable for the attachment of fibroblasts. Fayad et al [44] reported the complete absence of fibroblast attachment to root surfaces following the use of CO_2_ laser.

Some recent studies indicated that the application of Er:YAG laser significantly improves a number of clinical parameters [17,37, 38]. Dilsiz et al [3] discussed that the application of Er:YAG laser for root biomodification does not improve the results of SCTG. Bouchard et al [47] and Caffesse et al [18] reported that root surface conditioning with citric acid has no effect on the clinical results of SCTG. Our findings were in agreement with their results.

Further clinical studies are required to confirm the results of this study in larger study populations with longer follow-up periods.

## CONCLUSION

According to the results of this study, root surface conditioning by Er,Cr:YSGG laser improved the mean root coverage and the percentage of complete root coverage. However, these changes were not statistically significant.
